# GP perceptions of community-based children’s mental health services in Pennine Lancashire: a qualitative study

**DOI:** 10.3399/bjgpopen20X101075

**Published:** 2020-09-02

**Authors:** Alice Kate Lambert, Alison Jayne Doherty, Neil Wilson, Umesh Chauhan, Dushyanthan Mahadevan

**Affiliations:** 1 Child & Adolescent Mental Health Services, Bradford District Care NHS Foundation Trust, Saltaire, UK; 2 Faculty of Health & Wellbeing, University of Central Lancashire, Preston, UK; 3 Faculty of Health & Wellbeing, University of Central Lancashire, Preston, UK; 4 Faculty of Clinical and Biomedical Sciences, University of Central Lancashire, Preston, UK; 5 East Lancashire Child and Adolescent Services (ELCAS), East Lancashire Hospitals NHS Trust, Burnley, UK

**Keywords:** Mental health, Child health, Mental health services, Primary health care, Referral and consultation

## Abstract

**Background:**

GP satisfaction with specialist Child & Adolescent Mental Health Services (CAMHS) is often reported as low in the UK, and internationally.

**Aim:**

To explore GP perceptions of local children’s mental health services and to understand their experiences of a novel GP-attached Primary Mental Health Worker (PMHW) service.

**Design & setting:**

Qualitative research involving GPs in Pennine Lancashire.

**Method:**

Semi-structured face-to-face interviews of GPs (*n* = 9) were carried out. Thematic analysis was undertaken.

**Results:**

Themes identified included: 1) The role of the GP: most GPs perceived their role to be signposting and referring patients with mental health issues to specialist services, rather than offering care directly; 2) Clarity on help available: GPs were unclear about specialist CAMHS referral criteria and alternative resources available. GPs experienced communication challenges with specialist CAMHS; 3) Getting advice and support: PMHWs enabled GPs to have informal discussions, and to seek advice about children. Some GPs felt they could recognise problems earlier and were able to access help more quickly; and 4) Development needs: some GPs felt they required increased training in supporting children with mental health problems, and identified a need for further collaboration with schools and specialist CAMHS.

**Conclusion:**

The study identified challenges that GPs face with accessing and utilising specialist CAMHS. GPs who had PMHWs based in their practices expressed increased satisfaction with these services. GP-attached PMHWs can potentially reduce the challenges faced by GPs in primary care by offering timely and accessible advice, and improving access to specialist CAMHS.

## How this fits in

GP satisfaction with children’s mental health services is often reported as low, with GPs reporting many barriers to accessing specialist help. There has been an increase in children’s PMHW provision in the UK with a view to improving access to effective support, in line with '*Future in Mind*'.^1^ There is little research on the impact of PMHWs working with GPs. This study explores GPs’ perceptions of children’s mental health services in Pennine Lancashire, and their experiences of a novel GP-attached PMHW service.

## Introduction

The prevalence of mental disorders in children and young people is increasing nationally and internationally. In the UK one in eight children (aged 5 to 19 years) have a mental disorder, in keeping with the worldwide prevalence of mental disorders in children and adolescents of 13.4%.^2,3^ This has implications for future adult mental health, as most adult mental disorders start in childhood.^4^ The rising prevalence and future implications highlight the importance of prevention and early intervention for child and adolescent mental health problems.^5^ There has been much attention recently on the role of schools in providing mental health support, both in British government policy and research studies.^5,6^

When a parent is concerned about their child’s mental health, they typically present to their GP.^7^ Most British children visit their GP at least once a year.^8,9^ GPs are trusted by families, who want and appreciate their input.^4,10^ Given GPs’ unique position, it is unsurprising that they make more referrals to specialist CAMHS than any other single referral source. However, the rates of rejection of referrals from GPs to specialist CAMHS are three times higher than all other referral sources.^11^

GPs are often unclear about specialist CAMHS referral criteria.^7^ GPs may be unaware of other services and resources available.^11^ GPs are frustrated by rejected referrals, as they do not think that they make inappropriate referrals.^7^ GPs identified a lack of providers of specialist mental health services as the biggest barrier that prevents the effective management of child and adolescent mental health problems.^4^ Some may lack confidence in their skills and competence in dealing with child and adolescent mental health problems.^7,11,12^

Specialist CAMHS in the UK are experiencing an increase in referrals and waiting times.^13^ *’* *Future in Mind* *’* aspired that, by 2020, services would be built around the needs of children, young people, and their families.^1^ Significant financial investment is needed to deliver new models of care, with the provision of CAMHS starting from a low base.^14^

*Together We Stand* was a Health Advisory Service review of CAMHS in England and Wales.^15^ This described a tiered model of CAMHS. In Tier 1 social, emotional, and developmental support is provided by professionals that work in universal services, for example, GPs and teachers. These professionals are not specialists in child and adolescent mental health, but they have regular contact with young people, can offer advice and support for less severe mental health difficulties, and can refer to more specialist services as required.^16^ PMHWs can provide support to professionals working in Tier 1 and interface with specialist CAMHS, while being embedded in the communities they serve.^17^

‘THRIVE’ is a framework for system change that was developed by the Anna Freud National Centre for Children, and the Tavistock and Portman NHS Foundation Trust. It is an approach to delivering children and young people’s mental health support and services in a way that is both person-centred and needs-centred.^18^ THRIVE identifies needs relating to children, families, and professionals ‘getting advice’ — needs that PMHWs have historically met.

PMHWs have roles in consulting with and training professionals working with children with mental health problems in Tier 1 settings (see Box 1). In addition, PMHWs can provide support to children and young people with lower-level mental health problems. With better support and access to CAMHS, it has been argued that more mental health problems could be managed in primary care, reducing the need for specialist referral and enabling specialist CAMHS to concentrate on the severe or complex cases.^7^

Box 1 **The roles of children's Primary Mental Health Workers^17^**

**Consultation:** supporting universal Tier 1 services in identifying and managing children’s mental health needs.

**Intervention:** brief direct interventions as well as joint working with professionals in Tier 1 (for example, in primary care).

**Training and supervision:** offering multi-agency programmes of education to improve and consolidate skills in Tier 1.

**Liaison:** promoting collaboration and partnerships between agencies to increase accessibility.

**Strategic planning:** informing child mental health strategy and supporting the development of inter-agency structures.

**Research and development:** identifying service needs across agencies with regard to children’s mental health.

Various models for the organisation of PMHWs have been identified in the literature.^16^ These include PMHWs based in specialist CAMHS, schools, and GP practices, with various levels of independence and integration. There is scarce evidence for the efficacy of a specific service model in CAMHS.^19^ Many children report that school is not an environment in which they feel safe to be open about their mental health concerns.^1^ There is significant potential for a GP practice to provide a less stigmatising environment than a mental health clinic.^1,10^

In 2016, a pilot PMHW team for children was established in Pennine Lancashire. This area comprises Blackburn with Darwen, and East Lancashire. The total resident population of Pennine Lancashire is over 531 000. PMHWs were employed by the local specialist CAMHS provider, but were based in specific GP practices across Pennine Lancashire. At the time this study was undertaken, PMHWs worked across six GP practices. The aim of the team was to identify and meet needs in community-based settings wherever possible, while improving access for those children requiring specialist help. The role of the PMHWs was to provide advice, consultation, and training to professionals, and carry out direct clinical work with children and families in primary care settings.

This study aimed to explore GPs’ perceptions of CAMHS in Pennine Lancashire and to understand their experiences of GP-attached PMHWs. GPs’ perspectives were captured at a time when this new PMHW service was being established in primary care.

## Method

### Study design

The study comprised qualitative research involving semi-structured face-to-face interviews with GPs, conducted in GP practice settings in Pennine Lancashire. The interviews took place from September to November 2018.

### Data collection

A pre-piloted study topic guide (Appendix S1) was developed by the research team (including a GP) for use in the interviews. The study topic guide was based on a previous (unpublished) pilot study conducted in 2016, prior to the introduction and establishment of the new PMHW service, and comprised questions about GPs’ perceptions and experiences of giving and getting advice about mental health in children, accessing support from CAMHS, and managing risk.

### Participants

Purposive sampling was used for GP recruitment. The sampling framework used was the East Lancashire GP Federation list. GP practices were contacted on behalf of the research team by the GP Federation via email in August 2018. Emails were sent to all 36 practices who were affiliated with this collaboration of GPs in East Lancashire. The total population served by this federation is over 200 000 in an area of low life-expectancy and health inequalities.^20^

The emails inviting local GP practice partners to participate in the study contained two attachments: a letter of introduction, and a participant information sheet containing study details.

GP practice partners were also purposively sampled from the six new PMHW host practices within Pennine Lancashire. They were contacted by email in the same period.

Twelve GP partners expressed an interest in participating in the study. Potential participants were asked to contact a University of Central Lancashire researcher (AD) to arrange a convenient date and time for obtaining consent, and for interview. The researcher sent follow-up emails to five GPs, and a final follow-up email to two GPs. Three of these 12 GPs indicated that they were unable to participate in the study due to time constraints and *'needing to put the patient first*'.

Nine GP partners from the English local authority Lancashire areas of Burnley, Accrington, Blackburn with Darwen, Oswaldtwistle, Rawtenstall, and Nelson participated in the interviews. (See Table 1.) GPs were reimbursed for their time involved in the study’s participation.

**Table 1.** Demographic characteristics of GP participants and their patient populations

**ID**	**Sex**	**Status**	**Index of multiple deprivation** **score for GP practice (2019)[Table-fn T1_FN1]** ^a^	**Size of practice** ^b^ ****	**Base practice for PMHWs**
**GP1**	F	Partner	36.3	Medium	No
**GP2**	M	Partner	42.3	Medium	Yes
**GP3**	F	Partner	46.4	Medium	Yes
**GP4**	F	Partner	46.4	Medium	Yes
**GP5**	F	Partner	37.5	Large	No
**GP6**	F	Partner	24.4	Medium	No
**GP7**	F	Partner	30.3	Small	Yes
**GP8**	M	Partner	33.1	Large	No
**GP9**	F	Partner	34.0	Large	No

a Information obtained from National General Practice Profiles (available at https://fingertips.phe.org.uk/profile/general-practice/data). England: 21.7 (average).

b Small = patient population size of up to 4000; Medium = 4500 – 8000; Large = 8000-16 000. NHS East Lancashire CCG: 7172 (average), Blackburn with Darwen CCG: 7363 (average).

Four of these nine GPs received the new PMHW service (GPs 2, 3, 4, and 7), and the remainder did not receive this service (GPs 1, 5, 6, 8, and 9). The interviews were conducted by an independent researcher (AD) who was unknown to the participants. All the interviews were digitally audio-recorded, supplemented by written field notes. The average length of the recorded interviews was 23 minutes. The recorded interviews were transcribed by members of the research team’s support staff with transcribing skills and experience.

### Data analysis

Two researchers independently analysed the interview transcripts using thematic analysis to identify emergent themes (Table 2 summarises the analytical process).^21^ Open coding of each individual transcript was undertaken independently by the two researchers to explore the data within and then between the individual transcriptions. Coding was done first by hand, then NVivo (version 12) software was used to support the data management process. Basic themes were extracted from the coded segments and further refined to explore emerging themes. The independent analyses were then compared by the two researchers, and agreement reached through discussion.

**Table 2.** Summary of thematic analysis approach^21^

Step 1: Familiarisation of data
Step 2: Initial coding
Step 3: Generating themes
Step 4: Validity and reliability of themes
Step 5: Defining and naming themes
Step 6: Interpretation and reporting

### Results

Four main themes arose from the analysis of the data^2^: the role of the GP;^3^ clarity on help available;^4^ getting advice and support;^5^and development needs. These themes are discussed narratively below:

#### 1. The role of the GP

GPs indicated that they felt they had an important role as a first port of call for patients. GPs felt constrained in what they could offer to patients and their families in short appointments. Therefore, many GPs saw their role as:

*​* *’* *signpost or referrers in this instance* *,* *rather than necessarily dealing with the problem themselves.* *'* (GP8)

However, GPs felt that sometimes responsibility for children’s mental health was left solely with them:

*​* *'* *Because I think that we feel that probably is a specialist area* *,* *and we should have access to refer people on for that.* *'* (GP5)

#### 2. Clarity on help available

A common theme discussed by GPs was feeling ‘stuck’. GPs felt this way for several reasons, such as not being aware of available services or referral criteria:

*​'One of the problems that you have is that you are not quite sure which category they fit into. So, you may send the referral to the wrong place.'* (GP1)

GPs wanted to be kept up to date with available services, in an accessible format like a website; they found leaflets and posters difficult to access when needed. They felt ‘stuck’ when referrals to specialist services were rejected:

*​'Because we don’t necessarily, or I don’t necessarily, know what alternative services, maybe, could support them or what treatments I might be able to offer.'* (GP6)

GPs expressed a need for increased lower-level support for children and young people’s mental health to prevent issues from escalating into more serious mental health concerns.

#### 3. Getting advice and support

Some of the GPs interviewed had PMHWs based in their practices:

*​* *'* *She is thoroughly, thoroughly helpful really. It’s been a huge relief to have something like that. Quite well supported and quite luckily so.* *'* (GP7)

GPs found informal case discussions valuable. Often, when they were unsure of the diagnosis or where to refer to, GPs felt that discussion with PMHWs supported appropriate referrals. They felt more likely to refer to the right service at the outset and this prevented delays in the referral process. Even when the PMHWs were not on-site, GPs valued knowing they had a relationship with a PMHW that they could contact. The appointment of PMHWs made GPs feel more supported and therefore able to manage cases in primary care:

*​'For us, we are lucky. We have got the* [PMHW] *upstairs. So, it is fantastic. We can just access very easily. We can refer easily. If I am not sure about a case and where they need to go to … It has made a massive difference. Massive difference. I can think of many patients who I would have really struggled to manage. Really struggled. I wouldn’t have known what to do really. Because we have got the service here it’s really helped them. And helped me … So, I can think of quite a few patients it has made a huge difference to their lives.'* (GP3)

GPs perceived that patients prefer to be seen in primary care rather than in specialist CAMHS:

*​'It works well because patients like to come here. They don’t like going to different clinics and things … As soon as they go to mental health clinic, they know that everyone’s calling them crazy. Whereas they come to GP practice nobody knows what you have come for. So, it doesn’t matter which worker you are coming to see within the health centre. Nobody knows exactly who you are seeing.'* (GP4)

#### 4. Development needs

GPs spoke about areas for further improvement, including collaboration involving specialist CAMHS and schools. GPs said that they often advised young people and their families to seek support from the school, and contacted schools when making referrals. GPs wanted a shared understanding of the roles of school, primary care, and specialist CAMHS:

**** *'I think this has to be done in a much more holistic way. Of course, resources have to be put in. But it should start in the schools. It is where young people and children spend most of their time. So, most of these things should start at the school level. So, every school should have access to somebody who is expert in mental health.'* (GP2)

GPs acknowledged capacity constraints in CAMHS and that all children with mental health problems could not be seen by specialist CAMHS. GPs wanted:

**** *’prompt and clear communication about what was happening. So, if you sent a referral it’s triaged, and it’s assessed that they are going to be seen. We do get those notifications through, but getting them through fairly quickly would be really helpful.'* (GP9)

They felt that there was insufficient communication with third sector services that offer lower-level support services. GPs mentioned that they needed more training opportunities in child and adolescent mental health:

*​'Although I wonder whether joint training would be useful, but a resource that you could come back to again and again might be more useful. Something we could dip in and out of … Which I think is much more likely to be taken up on if there is an online resource that you can access in that way. It would be useful.'* (GP8)

## Discussion

### Summary

This study describes the challenges GPs experience when working with children with mental health problems. The GPs interviewed often perceived their role as a referrer to specialist services, but they were not always aware of available services or their referral criteria. Therefore, GPs often felt ‘stuck’ in managing children’s mental health problems.

This study highlighted the differences that GPs felt that the new PMHW model made. GPs described positive effects on accessibility of PMHWs for advice and support. The study also indicated that some GPs perceived that advice from PMHWs improved their ability to manage children’s mental health problems. PMHWs enabled GPs to have informal discussions regarding CAMHS, and to seek advice about children and appropriate referral. GPs felt they were able to recognise problems earlier and were able to access specialist assessment within primary care. Participants reported that children with mental health problems can find it easier to attend GP practices than mental health clinics, in line with views expressed in ‘*Future in Mind*’.^1^

GPs wanted a better understanding of training opportunities and resources in children’s mental health. PMHWs can support the development of a shared understanding of professional roles (for example, the role of specialist CAMHS). This may help to improve satisfaction with pathways to care. However, GPs also identified a need for better access to support for lower-level problems. There are limits to what improved understanding can achieve in a fragmented and under-resourced network of services.

Recent transformations in mental health services over the past decade, including Improving Access to Psychological Therapies, have resulted in more mental health problems being managed by specialist practitioners. With the planned expansion of mental health workers in schools in the UK,^6^ it will be important that there is an ongoing recognition of the high prevalence of mental health problems in those presenting in primary care.^22^ GPs will continue to identify and support children with mental health problems. Specialist CAMHS can support the delivery of integrated care in local communities by collaborating with primary care providers.

### Strengths and limitations

GPs from a wide range of practices were invited to participate in the study. The GPs interviewed were restricted to those who came forward following these invitations, which were co-ordinated by a local GP Federation. While the sample size for this study was small, due to challenges involved in recruiting GPs, it did elicit rich narrative data with the use of face-to-face semi-structured interviews.

### Comparison with existing literature

GP perceptions of specialist CAMHS in this study were consistent with findings in previous studies. GPs expressed concerns about where to refer patients who do not meet specialist CAMHS criteria, and the high rates of rejection of referrals to specialist CAMHS. This is in keeping with other research findings.^7,11,12^ The present study found that GPs feel frustrated by rejected referrals, which is consistent with the findings of O’Brien *et al*.^7^ The study also found that GPs want more training in children’s mental health.^7,11^

In this study, GPs identified benefits from PMHWs working in primary care. Having these specialist workers embedded in primary care reportedly improved accessibility of advice and support for professionals, and supported appropriate referral. GPs highlighted how the appointment of PMHWs had improved the accessibility of services for children and young people.

### Implications for research

In this study, the appointment of PMHWs mitigated some of the challenges perceived by GPs in meeting the needs of patients in the context of under-resourced children’s mental health services. This adds to the limited evidence-base relating to the delivery of mental health services for children in primary care. GP-attached PMHWs are being established elsewhere in the UK, and there is a need to understand the impact of such services.

Qualitative research can be an important part of understanding stakeholder perspectives, and can help these be used to shape service design and delivery. Future qualitative research relating to this model of care could also include the views of a wider range of stakeholders, including children, parents, PMHWs, and other professional groups.

Although this study has indicated that GPs view this model of care positively, there is a need for further research on new models of care in CAMHS that incorporate analysis of clinical outcomes at patient and service-level; economic evaluation; and broader impacts on community health.
